# Real-Time People Re-Identification and Tracking for Autonomous Platforms Using a Trajectory Prediction-Based Approach

**DOI:** 10.3390/s22155856

**Published:** 2022-08-05

**Authors:** Alexandra Ștefania Ghiță, Adina Magda Florea

**Affiliations:** Faculty of Automatic Control and Computers, University Politehnica of Bucharest, 060042 Bucharest, Romania

**Keywords:** person re-identification and tracking, trajectory prediction, computer vision, machine learning, social robotics, pedestrian tracking

## Abstract

Currently, the importance of autonomous operating devices is rising with the increasing number of applications that run on robotic platforms or self-driving cars. The context of social robotics assumes that robotic platforms operate autonomously in environments where people perform their daily activities. The ability to re-identify the same people through a sequence of images is a critical component for meaningful human-robot interactions. Considering the quick reactions required by a self-driving car for safety considerations, accurate real-time tracking and people trajectory prediction are mandatory. In this paper, we introduce a real-time people re-identification system based on a trajectory prediction method. We tackled the problem of trajectory prediction by introducing a system that combines semantic information from the environment with social influence from the other participants in the scene in order to predict the motion of each individual. We evaluated the system considering two possible case studies, social robotics and autonomous driving. In the context of social robotics, we integrated the proposed re-identification system as a module into the AMIRO framework that is designed for social robotic applications and assistive care scenarios. We performed multiple experiments in order to evaluate the performance of our proposed method, considering both the trajectory prediction component and the person re-identification system. We assessed the behaviour of our method on existing datasets and on real-time acquired data to obtain a quantitative evaluation of the system and a qualitative analysis. We report an improvement of over 5% for the MOTA metric when comparing our re-identification system with the existing module, on both evaluation scenarios, social robotics and autonomous driving.

## 1. Introduction

The research work in the current context is allocating a lot of resources to the field of autonomous operating devices, given its vast spectrum of applications. As recent studies [1,2] proved that social robotics and self-driving cars may impact human lives both economically and personally, there is an obvious increase in interest in developing systems that can be implemented on autonomous platforms. A thorough analysis of circumstances, adaptability to situations and environments, safe and reliable behaviours, and fast decision-making, are a few of the capabilities that need to be investigated to obtain robust and stable systems.

The function of a social assistive robot [3] is to assist people in their activities by engaging in meaningful interactions. Whether we are referring to offering information, guidance, reminders, instructions, etc., or just keeping company and maintaining a simple conversation, a social robot must be able to correctly identify and recognise the person it interacts with. In the context of social robotics, a person detection and re-identification component is imperative. In order for a robot to be able to analyse and understand the behaviour of the people it interacts with, it must be able to precisely map and model their positions.

A self-driving vehicle must be capable of moving safely through its surroundings without human input. It should navigate with or without passengers between two locations. While moving, the autonomous car must ensure the safety of all participants in the traffic: passengers, other drivers in its proximity or nearby pedestrians. While some studies focus on traffic understanding and decongestion [4], other research projects focus on implementing systems with robust capabilities [5]. In order to build a stable system, a person tracking and trajectory prediction system is required, for the vehicle to be able to analyse and prevent risky situations.

A person re-identification system is assigning the same identifiers to the exact same people in a temporal series of images. The characteristics of such a component are not limited only to social robotics and self-driving car applications but can be extended to any autonomous device. Whether we are talking about autonomous vehicles, robots, surveillance or smart home environments, being able to detect and recognise the same person through a sequence of images as well as distinguish between multiple individuals is a key constituent.

The problem of person re-identification has a complex nature as the performance of such a system can be strongly impacted by multiple factors. To design a system that performs well in a variety of scenarios implies creating a mechanism that is able to overcome problems such as occlusions or appearance variation. These problems arise from natural circumstances such as people passing behind obstacles while moving, similar styles in terms of clothing and hairstyle or interactions between individuals. To handle these types of problems our proposed method uses additional information generated by a trajectory prediction system.

People trajectory prediction is the ability to analyse the behaviour of a person and deduce their future movements based on the observations. Although it may seem straightforward, modelling the complex process behind human behaviour is a difficult task. The trajectories followed by the people in the environment are influenced by the movement of the other people in the neighbourhood, obstacles in the environment and points of interest in the scene. Trajectory prediction systems are required to integrate multiple factors in order to obtain an accurate result.

In this paper, we introduce a real-time people re-identification system based on a trajectory prediction method. The goal of this research is to improve a standard online tracking technique that we use in a robotic framework by incorporating information from a trajectory prediction system. We propose a trajectory prediction system that includes both social influence and visual information from the scene to estimate future positions. We validated our approach by performing multiple experiments on two possible applications: social robotics and autonomous driving. In the context of a social robotics application, we integrated our system in the AMIRO [6] robotics framework to improve the general behaviour of the Pepper (https://www.softbankrobotics.com/emea/en/pepper/, accessed on 27 July 2022) robot. The AMIRO framework is a modular platform used to program any Robot Operating System (ROS (https://www.ros.org/, accessed on 27 July 2022)) compatible robot. In the context of an autonomous driving application, we validated our approach on self-acquired videos to simulate a real-life scenario. We reported the results for the re-identification component and the trajectory prediction component individually, on existing datasets and on streams of images acquired in real-time. We presented several examples from our experiments, scenarios where the proposed system is overcoming complex problems while existing online tracking techniques would not obtain the same accurate results.

## 2. Related Work

The problem of person tracking and re-identification is a complex problem that has been intensively researched for a significant amount of time [7]. One of the early approaches for person re-identification can be traced back to 1997 [8]. Traditional methods used feature extraction and feature matching for people re-identification, while more recent approaches use complex systems based on neural networks for more accurate results.

Conventional person tracking and re-identification methods were divided into tracking by feature extraction and tracking by prediction to generate the results. The systems used simple visual descriptors such as SIFT [9] or HOG [10], alongside Kalman filters [11] to be able to re-identify a person in a sequence of images. The systems use approaches based on the data association methods [12,13,14] to assign the correct identification numbers to the right people. Such systems are susceptible to variations and occlusions of the people, as their representation of the information is not powerful enough.

The current approaches propose complex systems to solve the problem of people re-identification. In research such as [15,16], the architectures integrate temporal data to identify the people, while systems such as [17,18] perform sophisticated feature matching for re-identification.

The general applications of people re-identification systems usually require real-time processing. Autonomous devices need low latency information to adjust their behaviour according to the changes in the environment. One of the popular systems for online people tracking is DeepSort [19,20]. It combines visual information extracted using a convolutional neural network with a simple position estimation technique. The advantage of this system is that it can perform multi-tracking in a very short period of time. Tracktor [21] is another system which has a satisfactory performance on online multi-tracking. The simple technique based on a regressor of an object detector makes it suitable for simple applications that cannot implement a training phase. The disadvantages of these techniques, especially for the Tracktor system, is that they require a high framerate of the images.

To design a robust real-time system for the problem of person tracking and re-identification, we connected the existing methods with a more informed trajectory prediction system. We combined the advantages of the DeepSort system with the advantages of a trajectory prediction system that integrates environmental information and social influence, for a more powerful technique. Our proposed system overcomes the limitations concerning small framerates, as the introduced trajectory prediction module can adjust the estimation of trajectories based on the speed of the target.

As trajectory prediction is a valuable method that improves user experience and safety when talking about autonomous devices, the subject is widely researched in the field of artificial intelligence.

Some of the first systems that were introduced to approach the problem of people trajectory prediction, such as [22,23], were generating the results only based on the individual movement of the tracked person. The complex nature behind the movement of a person is not only determined by the inner state of the individual, but is dependent on the interactions with the environment or with the other participants. Taking into account these factors, a person may adjust their trajectory to avoid a collision or to move alongside a group.

The pioneering work conducted in Social LSTM [24] was the starting point for many directions in the research of person trajectory prediction. The key point introduced in their system is the integration of a social pooling layer between multiple recurrent neural networks that corresponded to the participants in the scene. The idea was later developed and improved in many papers, such as [25,26,27], by combining different networks to increase the precision. To model the social influence, [25] introduced a generative adversarial network (GAN) in combination with a pooling layer, while [26] combined recurrent networks with deep neural networks. The main disadvantage of these systems is that the predictions do not consider environmental information, which can alter the motion of an individual.

Papers such as [28,29,30,31] moved a step forward and improved the results of previous systems by introducing scene information into their systems. By analysing and combining both social influences and semantic information about the scene, the movement of a person can be better understood and modelled for more accurate results.

More recent approaches, such as systems in [32,33], are using goal-based prediction methods that proved to be effective for trajectory prediction. These kinds of methods are sampling multiple possible goal candidates for a person and choosing the best fit from that set of candidates.

Our system is built upon the more classical approaches that use recurrent neural networks to incorporate social and environmental interactions. We use grid images to encode the semantic information and pass the information to a generative adversarial network with a social pooling layer to encode the social influence. The system is using visual data to generate future positions for an individual. The previously cited papers assume a top-down view understanding of the scene, which is not always available in autonomous operating platform scenarios. The main advantage of our proposed method is that the system can predict trajectories using images acquired from an eye-level point of view. The proposed method allows dynamic environments with changes in terms of angles and visual cues. Moreover, the system generates quick predictions, using only image information (pixel coordinates and semantic data).

## 3. Proposed Method

We propose a system that tackles the problem of real-time person re-identification and tracking by combining a standard technique for people tracking with a people trajectory prediction method. The people trajectory prediction method combines the inner behavioural data of the people with external contextual information to predict the future possible positions of the people in the images. The information generated by the people trajectory prediction method is then combined with the information generated by a standard tracking system to generate a more precise real-time re-identification system.

### 3.1. People Trajectory Prediction

The goal of the people trajectory prediction system is to estimate the future trajectories of the people based on the observed information. In our approach, the observed information of the people includes contextual information about the participants in the scene and the scene settings. The model we propose generates the predictions by considering a combination between the coordinates of a person (pixels in the image) and the scene where the coordinates were recorded:

(1) $$x_{i}=\left(coord_{x},coord_{y},image\right),$$

The trajectory of one person consists of a sequence of tuples, coordinates and corresponding images, with respect to time. The social information between participants is encoded using an additional layer in the network.

The problem formulation behind trajectory prediction is represented by the ability to estimate X_gt_ based on X_obs_. X_obs_ represents the observed trajectory of a person, from the moment of time 1 to o, where 1 denotes the first element of the sequence and o is the number of the observed positions. X_gt_ represents the real trajectory followed by the individual, from the moment of time o+1 to o+p, where p is the number of elements in the prediction horizon. Our system aims to generate X_pred_, an estimation of X_gt_, such that the errors are minimal.

**Figure 1:**
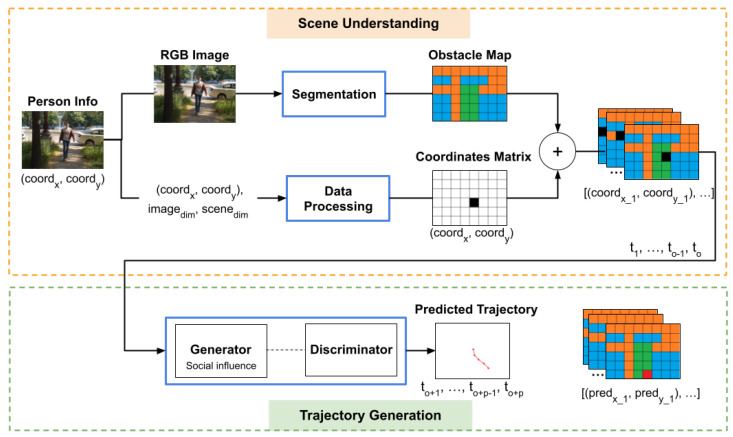
Social–GAN with Obstacle Map architecture. The aggregated data is computed for each person in the images.

The architecture of the system consists mainly of two modules: a module for scene understanding and a module for generating the predicted trajectory. The structure of the architecture is shown in Figure 1. The Scene Understanding module extracts the visual data associated with the input image, generating information about the scene, such as obstacles or pathways, alongside the corresponding position of the person. The Trajectory Generation module integrates the generated visual data to estimate the most plausible trajectory, based on the scene settings, the other participants and the movement of the person.

The proposed architecture integrates three factors that can influence the trajectory of an individual: inner behaviour, social influence and scene settings. The inner behaviour of a person refers to the previous positions in which the person was observed. In Figure 1, the inner behaviour is modelled by the 2D coordinates alongside the corresponding coordinates matrices. Social influence refers to the movement of the other participants in the scene, that can influence the trajectory of a person, by avoiding the intersection of trajectories. In the architecture, the social influence is encoded by a pooling layer in the Trajectory Generation module, which transfers information between the people in the neighbourhood. The scene settings are represented by the layout of the environment, in terms of obstacles and possible pathways. This information is encoded by the obstacle map, which is generated based on the segmentation mask of the input image.

The information associated with a person which is passed as input to the system is composed of a mix of the coordinates representing the position of the person at each time step, alongside the RGB images of the scene acquired at that exact time. Based on this information, the system computes two additional pieces of information: the pixel coordinates matrix of the person and the obstacle map of the environment.

#### 3.1.1. Obstacle Map

The obstacle map integrates the shape of the scene in terms of obstacles and walking areas. We use a segmentation network to determine all the object classes in the image. We applied a ResNet50 network [34] combined with a Pyramid Pooling Module [35] to compute the segmentation mask of the image. The ResNet50 network is a deep convolutional neural network that uses multiple residual blocks for the accurate classification of the images. The skip connections introduced by the residual blocks pass the information unaltered to deeper layers to reduce the errors caused by vanishing gradients. The Pyramid Pooling Module [35] is used to further improve the overall performance of the Resnet by adding more information into the network, considering different scales and sub-regions from the images. The network was trained on MIT ADE20K scene parsing dataset [36,37] as it includes classes of objects that can be found both indoors and outdoors. As a trajectory prediction system can be utilised in multiple scenarios, such as an indoor assistive robot or an autonomous car, the segmentation mask should be able to detect the obstacles in all possible areas.

As the dataset used for training the segmentation network has a large number of classes, we simplified the segmentation mask by grouping the classes into three main classes: obstacle, preferable walking area and possible walking area. The segmentation network will generate an output for an image considering only these three classes. To eliminate the problem of different image sizes between datasets, we applied a transformation of the image into a grid matrix. We split the segmentation mask into a fixed number of grids on both axes, horizontally and vertically, and we assigned each grid the value of the class with the highest number of appearances in that patch.

#### 3.1.2. Pixel Coordinates Matrix

The pixel coordinates matrix consists of a grid representation of the position of the person in the image, according to the obstacle map. Our method uses the coordinates expressed in pixels to predict future trajectories. Some of the datasets integrated into the validation phase do not contain the image positions of the people, but only include the real-world coordinates relative to the scene (values represented in meters). To be able to apply the proposed method to those specific datasets, we applied an extra processing step to derive the required pixel information, based on the proportion and correlation between real coordinates and the people in the image. We determined the size of the area that is visible in the images by computing the values of the width and height expressed in meters, based on the coordinates of the people that appear on the edges of the images. We estimated the pixel coordinates considering the following formulas:

(2) $$coord_{x}=\left(\left(scene_{x}+displacement_{x}\right)/scene_{width}\right)\times image_{width}$$

(3) $$coord_{y}=\left(\left(scene_{y}+displacement_{y}\right)/scene_{height}\right)\times image_{height}$$

The variables *displacement* on the axis *x* and *y* are used in the cases where the origin of the scene, based on which the real-world coordinates are computed, is not visible in the image, so a translation of the coordinates is required.

To have homogeneous dimensions between all the datasets used in the training phase regardless of the size of the images, we compute a squared grid matrix for each image. We split the original image into a fixed number of grids (horizontally and vertically) and we assign 1 for the grid where the person is located and 0 in the rest, according to the following formulas:

(4) $$grid_{x}=coord_{x}/\left(image_{width}/grid_{size}\right)$$

(5) $$grid_{y}=coord_{y}/\left(image_{height}/grid_{size}\right)$$

#### 3.1.3. Social-GAN

The Trajectory Generation module is processing the observations generated by the Scene Understanding module, to generate the most plausible future trajectory. The observed data consists of a sequence of consecutive obstacle maps, coordinates matrices and corresponding coordinates, combined into a single volume input, which is passed to the Social-GAN component.

The structure of the Social-GAN component is similar to the architecture presented in [25]. We use a generator-discriminator structure to predict the trajectory of a person. The generator uses a pooling module placed between an encoder and a decoder to integrate social information about the movement of the people in the neighbourhood. The social information is represented by the coordinates of the other participants in the scene relative to the person, encoded by a Multi-Layer Perceptron. The pooling layer exchanges this information between all the participants in the environment. The main difference between the system in [25] and ours is that our system predicts obstacle maps alongside the trajectory coordinates for a person. The output of the generator includes the predicted obstacle maps corresponding to each predicted position in the estimated trajectory, to be able to penalise the incorrectly placed coordinates. The loss function used to train the network is obtained by combining the errors obtained on the trajectory coordinates and the errors of the estimated positions of the person on the predicted obstacle maps.

### 3.2. Real-Time People Re-Identification and Tracking

The end goal of our system is to be able to track and re-identify people in an image stream. The system needs to be able to recognise (re-identify) the people by associating the same numbers to the exact same persons through a succession of images.

**Figure 2:**
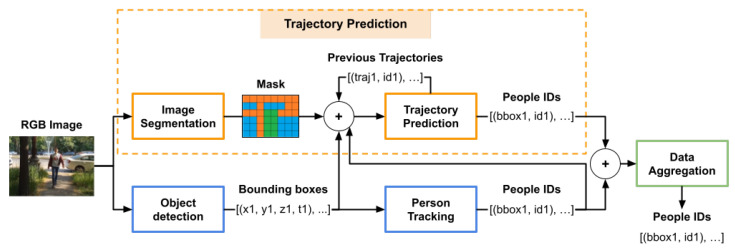
Real-time Person Re-identification and Tracking architecture based on a People Trajectory Prediction component.

The architecture we propose takes as input a stream of images and predicts an identifier number for each person detected in the image. Figure 2 presents the layout we designed for the real-time re-identification system. The proposed architecture can be integrated into both a robotic platform system and an autonomous vehicle as it requires only RGB data to generate results.

We combined five modules for the real-time person re-identification task: object detection, person tracking, image segmentation, trajectory prediction and data aggregation.

The object detection module generates bounding boxes representing the positions of the people for each acquired image. There are multiple networks that obtain accurate results for the task of object detection. In our real-time experiments, we used the YOLO architecture [38], as it matches our performance and speed requirements. For the rest of the experiments, we handled the pre-computed detections available inside the datasets we used.

The person tracking module is a standard tracking technique that computes identification numbers for each person in the images. It uses the system introduced in [19,20]. It combines a simple technique for estimating future positions with a convolutional network that computes deep visual features for the detected people. The module generates real-time results for the task of re-identification.

The image segmentation and trajectory predictor modules are the constituents of the person trajectory prediction method described in Section 3.1. The image segmentation module computes fairly accurate results in a short span of time, necessity introduced by the real-time requirement of the application. However, to further reduce the computation time in real-time scenarios, the segmentation module is run in parallel with the object detector, as shown in the architecture in Figure 2.

The trajectory prediction module generates future possible positions for the people tracked in the images. To compute the results, it requires sequences of consecutive observed positions of the people in the images, alongside visual data from the scene. The required positions are determined by the bounding boxes extracted by the object detector and the environmental information is extracted by a semantic segmentation network. In the real-time re-identification architecture the trajectory prediction module requires additional information to be able to generate trajectories. For the initial observed positions of the people, the module uses the data generated by a tracking predictor, and for the rest of the process, it completes the missing information with the previously generated results.

For each image acquired by the autonomous platform, the system computes the identifiers and the future trajectories for the detected people. To transform trajectories into identification numbers, we take into consideration the current people detections (bounding boxes) and the trajectories predicted at the previous frame. Each future trajectory generated by the module in the previous step is associated in the current step with one of the bounding boxes extracted by the object detector. The association is achieved by computing the Euclidean distances between the positions of the bounding boxes and the first estimated future position of the trajectory. If the minimum Euclidean distance is within a predefined threshold, then that person, who is represented by the closest bounding box, is associated with the identification number which corresponded to that particular trajectory at the previous step. If there is a trajectory that has no corresponding bounding box, which may be the case in which a person is occluded, then the module completes the current information and adds as the current position of the occluded person, the estimated position from the previously predicted trajectory. By completing the information for the occluded people with the estimated positions, the module can still predict their future possible positions, and when the people are no longer occluded it can re-associate their correct previous identifier.

The data aggregation module combines the results obtained by the trajectory prediction and person tracking to generate more accurate re-identification IDs for the people in the images. Each of the two modules, trajectory prediction and person tracking, estimate separate identification numbers for the people in the image at a moment of time. The data aggregation module computes the final IDs based on the confidence and results of the two modules. With every image, the trajectory predictor gains more confidence as it acquires more information about the previous trajectories. In the case that the trajectory predictor fails to associate feasible IDs to the detected people, the estimated IDs are exclusively the ones obtained by the person tracking module.

## 4. Experiments

We tested and analysed the performance of the proposed system in various scenarios and applications. The purpose of our project is to implement the re-identification system on an autonomous platform, whether we talk about robots or self-driving cars. All the executed experiments were conducted from the point of view of an autonomous platform. The design of the architectures and the processed results were obtained considering the real-time requirements of such an application. We presented the results considering the two possible use cases: social robotics and self-driving vehicles. We divided the experiments into two parts, to evaluate objectively and independently the performance of the full re-identification system in different scenarios and the performance of the trajectory prediction on existing datasets.

### 4.1. People Trajectory Prediction

We analysed the people trajectory prediction component considering the two case studies relevant for our project, from the point of view of a robot and from a point of view of an autonomous vehicle. The network was trained based on the corresponding presented datasets, using observation and prediction sequences of length 8. The network was trained for 200 epochs, using a global pooling mechanism. For both case studies, the errors reported for one subset were obtained by training the network against the rest of the subsets.

#### 4.1.1. Validation in the Context of Social Robotics

The context of social robotics assumes interactions between people and robots for meaningful interactions. The re-identification system is a key component for a robotic platform to exhibit a precise behaviour. The information generated by the trajectory prediction module has two objectives: one is to generate accurate tracking identification numbers for the detected people, and one is to help the robot to be able to meet or avoid the people in the environment.

To obtain a more precise evaluation of our system with respect to the end goal of a social robotics application, we used the JRDB [39] dataset. The JRDB dataset is a data collection of images acquired by a robotic platform, in both indoor and outdoor environments. It contains multiple subsets, with different scene settings, various lighting and different numbers of participants. We chose to integrate this dataset into our evaluation because it simulates conditions closer to the real ones. The robotic platform used to acquire images moves through the environment and some people stop to interact with it.

**Figure 3:**
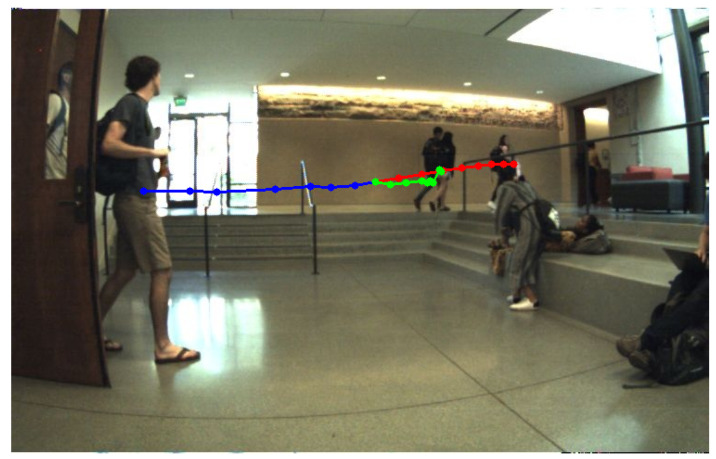
Visualisation of a predicted trajectory based on previous observation in an image extracted from the JRDB [39] dataset. The blue dotted line represents the observed positions of the person, the green dotted line represents the real positions followed by the person, and the red dotted line represents the future predicted positions estimated by our system.

Figure 3 presents an example trajectory generated based on a sequence of images extracted from the JRDB [39] dataset. In the image, the blue dots represent the observed trajectory of the person, the red dots represent the trajectory predicted by the system and the green dots represent the real trajectory followed by the person. The example image that is presented is acquired in an indoor environment, from the point of view of a robotic platform. The person tracked in this scenario walks from the door on the left of the image to the door on the right of the image. The trajectory predicted by our system is predicting the correct direction, with a higher moving speed than the actual speed. Even though the person is following the same predicted path to the right door, we can observe a higher reduction in the speed when reaching the stairs than the one anticipated by our system.

Table 1 presents a detailed report of the results obtained by the system for the subsets contained in the JRDB dataset. The values represent the errors obtained at the testing phase for one subset while training the system on the rest of the subsets in the dataset. We only considered the datasets that included 3D information about the people in the images, besides the position of the bounding boxes. The table presents the values in pixels obtained for two metrics, ADE, average displacement error, and FDE, final displacement error. The metrics compute the difference between the estimated pixel coordinates and the actual coordinates of the people in the images. The average displacement error is computed as the average of the root mean squared error between every point in the predicted trajectory and every point in the ground truth trajectory. The final displacement error is computed as the Euclidian distance between the final points of the predicted trajectory and the ground truth trajectory. The metrics were reported for predicted trajectories of length 8. Each subset was pre-processed to simulate a frame rate of 2.5 frames per second. The size of the images is 752 × 480 pixels. The subsets in the table are grouped into two classes, corresponding to the two possible scene settings, indoor and outdoor. As it can be observed, there is no significant difference between the results of indoor and outdoor subsets. What can be noticed is that in some subsets the values of the errors are very small, with a final displacement error of 7 pixels, while others have a final displacement error of around 60 pixels. The difference comes from the image conditions of the subsets. For example, in the case of the cubberly-auditorium_0 subset, the images include a lot of people that are being tracked while the robotic platform is moving through the environment. This particular case makes it difficult for a more accurate trajectory prediction, as the ego-motion of the platform needs to be taken into consideration. Regardless of the type of scene, the average error values obtained for the trajectories are small enough for a satisfactory behaviour of the robotic platform.

The values reported in Table 1 and the example represented in Figure 3 present the errors obtained between the estimated and ground truth trajectories as pixel positions in the images. Classical trajectory prediction methods report the results obtained based on the real position in the scene. To be able to position our work with respect to other existing methods, we also included in our evaluation the two classical datasets for trajectory prediction, UCY [40] and ETH [41]. UCY [40] and ETH [41] datasets are data collections that contain tracking data about the people in the images as real positions in the scene, but without the pixel positions in the images. The datasets also contain the associated images which are acquired from a bird’s-eye point of view. As the datasets include only the information regarding the world coordinates and associated images, they were pre-processed in order to estimate the positions of the individuals in the images based on the world coordinates, in order to generate the grid matrices. To have an objective comparison we considered the same two scenarios for trajectory prediction as in previous work [25,28]: we observe 8 positions for a person and predict the trajectory for the next 8 and 12 positions. We used the same architecture to predict the world coordinates with the same principle of obstacle maps by estimating at each moment of time the position of the world coordinates on the obstacle map. The results of our experiments in relation to other systems are reported in Table 2. Each cell contains the two error values expressed in meters for the prediction of 8 positions, respectively, 12 positions.

Even though our method is not designed to improve the world coordinates trajectories, we performed the comparison to position and assess our method with respect to the other existing systems in trajectory prediction. We can observe that the system obtains smaller errors on the UCY [40] when compared to the ETH [41] dataset. The difference comes from the results of the segmentation technique applied to the images. The segmentation network we used is specialised in eye-level view images, and when applied to bird’s-eye view images it is not as accurate. In particular, for the ETH dataset the results of the segmentation technique are not as good as in the case of the UCY dataset. For the UCY dataset, we obtain similar results to the existing techniques.

#### 4.1.2. Validation in the Context of Autonomous Driving

The context of autonomous driving implies detecting pedestrians on the side of the road and estimating their future positions. Trajectory prediction is a very important component for a self-driving car as it needs to understand the movement of the pedestrians to be able to safely move past them.

To have an objective evaluation of the people trajectory prediction component for self-driving cars we used the Caltech-Pedestrians dataset [42]. Caltech-Pedestrians is a data collection that includes 11 subsets of videos acquired from a moving car in an urban environment at a framerate of 30 frames per second. As the trajectory prediction needs 8 observed positions to predict the future trajectory, a high framerate is not suitable for our system. To observe the movement of a person for less than 300 milliseconds is not informative enough to make a reliable prediction. Consequently, we applied a pre-processing step to simulate a lower framerate of the videos in the dataset.

**Figure 4:**
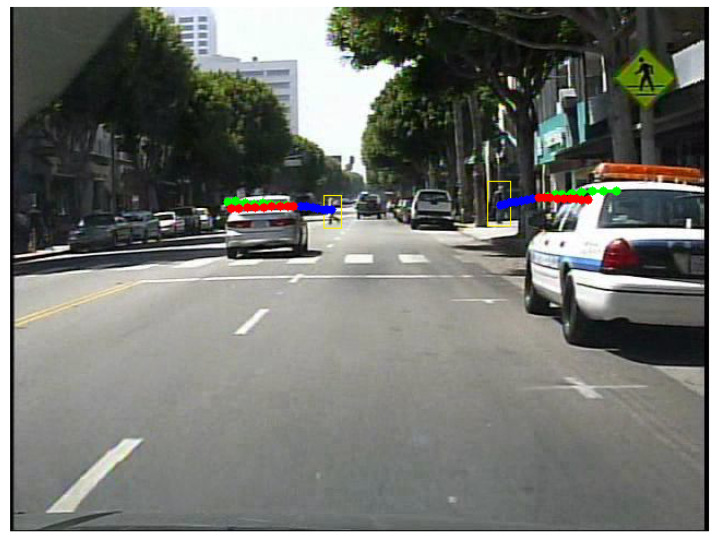
Visualisation of two pedestrians predicted trajectories based on previous observation in a sequence of images extracted from the Caltech-Pedestrians [42] dataset. The blue dotted lines represent the observed positions of the people, the green dotted lines represent the real positions followed by the people, and the red dotted lines represent the future predicted positions estimated by our system.

Figure 4 shows an example of predicted trajectories in a sequence of images extracted from the Caltech-Pedestrians dataset. The blue dots represent the observed trajectories of the people, the green dots represent the real trajectories followed by the people and the red dots the estimated trajectories. This particular example demonstrates the performance of the system when the camera is not static. In this video, the vehicle is moving forward, and it can be noticed that both trajectories are predicted correctly, in both directions.

Table 3 presents a more detailed view of the performance of the system in a self-driving car context. We computed the ADE, average displacement error, and FDE, final displacement error, for each subset included in the Caltech-Pedestrians dataset, as previously performed in Section 4.1.1. The error values are reported in pixels, for observed and predicted trajectories of length 8.

The errors reported in Table 3 prove that there is no significant difference between the subsets. The only FDE that has a slightly higher value compared with the rest of the subsets is obtained for set00, but the ADE for the same subset is comparable with the other values. The evaluation of the system obtained small errors for the trajectory prediction component, with the highest FDE of 21.35 pixels and the smallest FDE of 7.20 pixels, considering that the size of the images is 640 × 480 pixels. The Default split line presents the ADE and FDE values obtained using the split point for the train-test suggested by the authors in [42]. The recommended training subset is composed of the subsets set00 to set05 and the testing subset consists of the subsets set06 to set10. The values do not diverge from the computed average errors, proving that the functioning of the system is robust and reliable.

### 4.2. People Re-Identification and Tracking

The real-time people re-identification and tracking component is evaluated on the same case studies relevant for our project, social robotics and autonomous driving. Parts of the information presented in Section 4.2.1, namely processing time, experiments settings, and missing information approach, are also applicable for Section 4.2.2. The evaluation of this component is also performed on an additional use case, considering images acquired from a bird-eye view angle, to demonstrate the generalisation of the solution.

#### 4.2.1. Validation in the Context of Social Robotics

**Figure 5:**
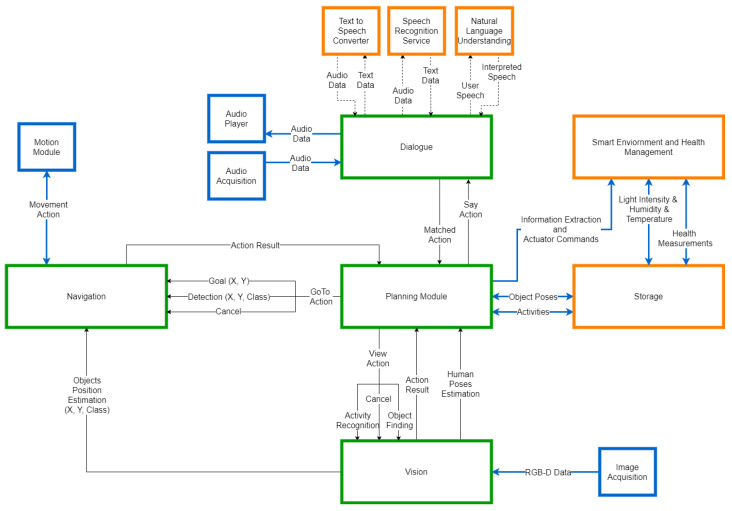
Architecture of the AMIRO framework for Social Robotics [6]. The green boxes correspond to cloud-egde components, the orange boxes correspond to cloud services, while the blue boxes represent the modules running on the robot.

To evaluate the results considering a social robotics application, we integrated the system into the AMIRO framework [6], a platform for operating robots in the context of social robotics, with the goal of improving the performance of the general behaviour of the Pepper robot. The basis of our integration was performed considering the architecture of the framework presented in Figure 5.

The architecture of the AMIRO framework, as presented in Figure 5, is built as a composition of independent modules which operate in parallel. The data generated by each main component is processed independently and then sent to the Planning module. Our proposed system is integrated inside the Vision component of the AMIRO framework and is generating identification numbers associated to each person in an image. The aim of our system is to re-identify the people in a sequence of images, by associating the same numbers to the exact same persons. The re-identification by identifier of a person is more reliable than recognition by face, as it does not require face visibility and recognition during the full interaction. The improvement of the re-identification component should considerably improve the general behaviour of the robot, considering sub-modules such as activity recognition or object finding.

Considering Figure 2, the integration inside the AMIRO framework is straightforward. The images passed to our proposed system are acquired from the robot by the Image Acquisition module. We use the existing detection module inside the Vision component to compute the bounding boxes of the people, and we associate each bounding box an identification number generated by our proposed system. The rest of functionalities of the AMIRO framework continue to operate in the same manner, with the improvement of recognising the people by identification number.

One of the objectives of our project is to improve the robustness of the behaviour of the Pepper robot in the context of social robotics applications. One of our goals is to design a robust architecture that can successfully tackle the challenges of ambient assisted living. We intend to use the Pepper robot in the kind of applications where the environment is not very crowded, in such a manner that the robot can focus its attention on one person at a time, for meaningful interactions. The robot may interact with a person at home or can also function as an information point or a guide in a museum. Considering the possible scenarios for such a robot, we evaluated the proposed tracking and re-identification system in real-time by reproducing the same potential conditions. We analysed the results obtained by the system in different environment settings. The system was tested both in indoor and outdoor environments, with one or multiple people in the images.

The experiments we performed tested various scenarios that can appear while a robot tracks a person. The scenarios vary in terms of environmental conditions, such as background, light, distance or obstacles that may cause full or partial occlusions, but also in terms of movement of the people, such as interactions, occlusions by movement, sudden change in the movement of the people.

**Figure 6:**
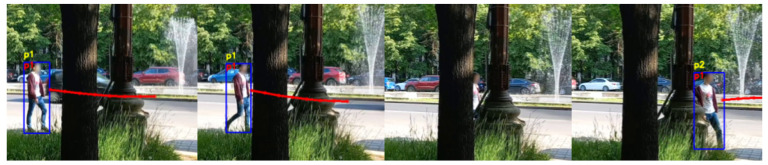
Person re-identification based on trajectory prediction in the case of a person being fully occluded for a sequence of images. The yellow ID represents the identification number estimated by the standard tracking technique, and the red ID represents the one generated by our system.

**Figure 7:**
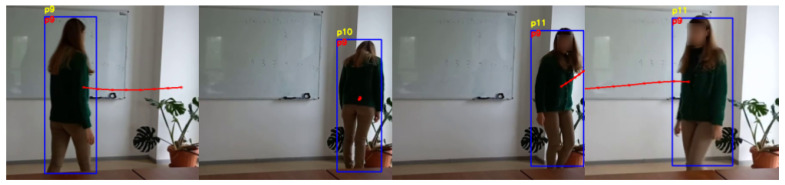
Person re-identification based on trajectory prediction in the case of a person changing the initial direction. The yellow ID represents the identification number estimated by the standard tracking technique, and the red ID represents the one generated by our system.

**Figure 8:**
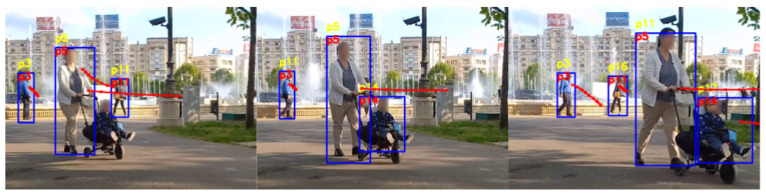
Person re-identification based on trajectory prediction in the case of people overlapping. The yellow ID represents the identification number estimated by the standard tracking technique, and the red ID represents the one generated by our system.

**Figure 9:**
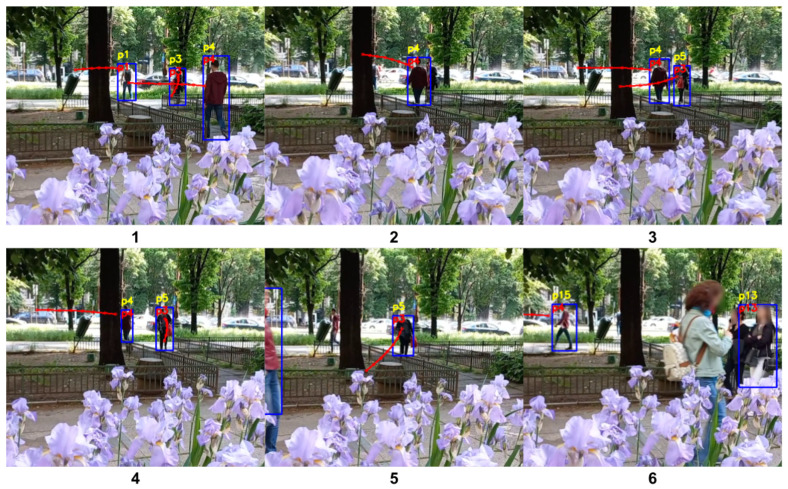
Person re-identification based on trajectory prediction in the case of people overlapping and occlusions generated by the environment. The yellow ID represents the label estimated by the standard tracking technique, and the red ID represents the one generated by our system.

Both the standard tracking and our trajectory prediction-based tracking handle simple circumstances well, with no switch of the identifiers for the tracked people. The situations that involve no occlusions or no change in the visual appearance of the people are not difficult to manage by both systems. However, the current tracking system used in the AMIRO framework exhibits problems when a person is occluded for a number of frames, or the visual cues are changing. Figure 6, Figure 7, Figure 8 and Figure 9 are examples of situations where the standard tracking technique is not performing as expected. In all four figures, the blue rectangles represent the bounding boxes of the detected people generated by the object detector module, the yellow number above a bounding box represents the re-identification number associated by the standard tracking technique to a person and the red number inside a bounding box represents the re-identification number associated with the proposed system. Every reported frame also contains the expected future trajectories for the people that were observed for a minimum number of positions. The figures contain only a subset with the relevant images from the full experiment.

Figure 6 shows an example of a person that is being fully occluded for multiple consecutive frames. The person is moving from the left of the image to the right while passing behind an obstacle. The person, who initially is identified as *p1* by the standard tracking system, is switched to *p2* when re-detected after the missing frames. Our system corrects this behaviour and assigns the same identifier *p1* despite the missing detections. The predicted trajectory associated with the person before the occlusion, which is shown in the second image of Figure 6, was estimated that *p1* will go straight and will pass the obstacles. When the person was re-detected, as the detection matched the estimated position at the correct time, it was assigned the same identification number.

Figure 7 presents a scenario where a person is going to a particular location then changes the direction and returns to the initial position. This scenario produces sudden changes in the trajectory followed by the tracked person and in the visual information extracted from the person. In this scenario, the person, who is going to the plant on the right of the image and then returns, is initially assigned the identification number *p9*. The standard tracking technique switches to *p10* when the person stops moving and leans towards the plant, and switches again to *p11* when the person rotates and faces the camera. Our proposed system overcomes these problems and keeps the same initial identifier, *p9*, for the whole sequence of images. The images also present the predicted future trajectories during the experiment, for a better understanding of how they dynamically adapt.

Figure 8 and Figure 9 present images from experiments we performed with multiple people in the images. In the images, as the people are moving considerably, there are occlusions and variations in trajectories and appearance.

Figure 8 is an example of an experiment with four tracked people. The people *p3*, *p11*, who are in the back of the image, and *p5* are initially tracked correctly by the standard tracking system. When target *p11* is no longer visible as it is being occluded by *p5*, the tracking system is switching the identifier for *p3* to *p11*. When *p11* becomes visible again, the identifier for *p3* switches to the initial value of *p3*, *p5* switches to *p11* and *p11* is assigned a new identifier, which is *p16*. Moreover, the baby in the cart, which initially is assigned the identifier *p14*, is switched to *p17* while moving to the right of the image. The performance of the tracking system in this scenario would extremely impact the performance of a robotic system, as it would not be able to focus on a person properly. Our trajectory prediction-based system correctly re-identifies all the people in the scenario, regardless of being occluded or not. As the trajectory for target *p11* was estimating that it will move towards the initial position of *p5* after a number of frames, when it was re-detected, it was correctly re-assigned the same identification number. The predicted future trajectories for *p3*, *p5* and *p14* offered the system the information needed to maintain the right identification numbers for the people during the experiment.

Figure 9 presents another example of multi-tracking. This particular example presents how the system performs for a longer and more complex scenario. Person *p4* is walking towards the left of the image, after avoiding a tree. While *p4* is following this route, a group of people is passing in front of the camera covering *p4* for a number of frames. In this scenario, *p4* is occluded by both an obstacle and other participants in the environment. The standard tracking system is initially assigning the correct identification number when intersecting with *p3*, but after being occluded for multiple frames, the system switches the ID to *p15*. Moreover, we can observe that person *p3* is changed to *p5* after intersecting with *p4*. Our proposed system correctly tracks both *p3* and *p4*, given the predicted trajectories. This example also shows how the trajectories of the people are adapting with every detection, from initially going towards the centre of the image for person *p4*, to then adjusting the trajectory towards the left of the image.

The accuracy of the full re-identification component is strongly dependent on the other components of the AMIRO framework: image acquisition, object detection, tracking. The Pepper robotic platform we use provides images at a resolution of 640 × 480 with a varying framerate. To have a more objective assessment of the performance of the system we used the MOT17 [43] dataset to compute [44] several tracking metrics. The MOT17 dataset contains images acquired from an eye-level point of view in multiple environments, both indoor and outdoor. We used this dataset as it is the most referable when talking about people tracking. The metrics we computed are MOTA (Multiple Object Tracking Accuracy) [45], IDF1 (Identification F1) [46] and HOTA (Higher Order Tracking Accuracy) [47]. Table 4 reports the tracking values obtained based on the available detections files computed using the SDP [48] network. The proposed system obtains a higher value for the MOTA metric and similar values for IDF and HOTA. These values were obtained by using a trajectory prediction model trained on the JRDB dataset. The JRDB dataset was pre-processed to simulate a framerate of 2.5 frames per second, to match the framerate of our robot. If trained on the MOT17 dataset, using the correct framerate as the subsets in MOT17, we expect the results to be better.

Table 5 presents the mean processing time of the system considering a different number of tracked people. The time is measured from the moment when the trajectory prediction module receives the input until the moment the data aggregation module generates the result. As it can be observed, the system obtains similar speed values regardless of the number of people being tracked. This proves that the system can perform multi-tracking in the same processing time as it would require for single-person tracking. Moreover, the processing time is reasonably small, making it suitable for real-time applications.

All the experiments we performed used trajectory predictions of eight future positions. We decided upon this constant by taking into consideration the framerate of the Pepper robot and the observations we made on how often people change the apparent trajectory. The network we trained and integrated into the AMIRO framework predicts trajectories based on observations of length 8. Considering that the Pepper robot integrated into our AMIRO framework has a smaller framerate, to be able to estimate trajectories quickly we decided to initially simulate the observed trajectory of a person starting from three observed positions. When a person is observed for three consecutive frames, we fill the rest of the missing data with the first detected position, creating a trajectory that stays on the same first observed position for six frames, then goes to the direction represented by the last two observed positions. As the system receives more and more data, for every detection received by the system for a person, one of the simulated positions is removed. Using this mechanism, the system can generate trajectories faster and it does not require waiting for eight consecutive detections for the same person to generate a trajectory. The reported results include the speed-up technique.

The system tackles the problem of missing continuous observations by using the positions of previously predicted trajectories. If a person that was being tracked is not detected in a frame, it is assumed that the person is occluded and that the current position of the person is coinciding with the first position of the predicted trajectory. This method allows the system to complete the information for eight missing frames which correspond to the eight predicted future positions. If the person is not re-identified during eight frames, then the information associated with that person is removed. We added several videos (https://drive.google.com/drive/folders/1jjOmzQQH2n5AlPhdXTYohvkoNgSh3Kun?usp=sharing, accessed on 27 July 2022) to prove the performance of our system.

#### 4.2.2. Validation in the Context of Autonomous Driving

Re-identification and tracking in the context of autonomous driving is a more challenging problem than in the context of social robotics. To be able to correctly re-identify a person through a succession of images, the system requires a model that can encode camera movement, scale variation and multiple occlusions. The movement of the camera is an important constraint in the problem of re-identification, as it implies not only changes in the angle views, but also fluctuations in the speed of movement. The fluctuations that appear in terms of movement speed can significantly influence the physical appearance of a person when considering scaling or observed positions. These limitations are not a relevant factor in the context of social robotics, as usually, the robots move slowly through the environment for safety reasons.

**Figure 10:**
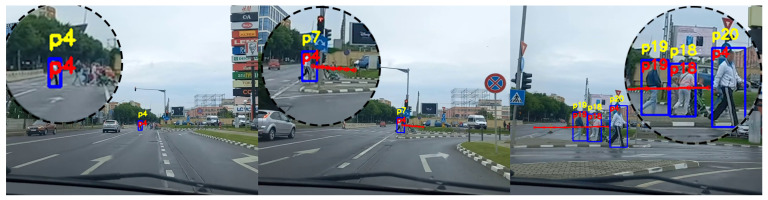
Person re-identification results in a scenario where the car is moving right and the pedestrians are crossing the street. The yellow ID represents the identification number estimated by the standard tracking technique, and the red ID represents the one generated by our system.

**Figure 11:**
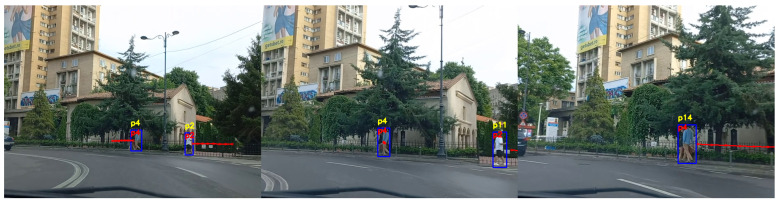
Person re-identification results in a scenario where the car is turning left and the pedestrians move on the sidewalk. The yellow ID represents the identification number estimated by the standard tracking technique, and the red ID represents the one generated by our system.

**Figure 12:**
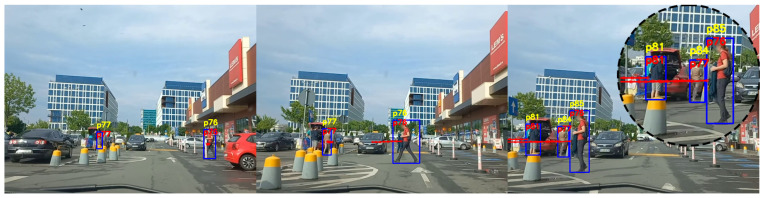
Person re-identification results in a scenario where the car is moving straight and the pedestrians are in front of the car. The yellow ID represents the identification number estimated by the standard tracking technique, and the red ID represents the one generated by our system.

The experiments we performed proved that the standard tracking system is very susceptible to errors when tested on videos acquired from a moving car. The tracking system has an acceptable performance in situations where the car moves very slowly and the pedestrians are close to the camera. However, there are a lot of identity switches when the car moves at a normal speed, or the pedestrians are at a greater distance, or the car is turning and is changing the angle view of the camera. Figure 10, Figure 11 and Figure 12 are three examples extracted from our experiments to demonstrate the performance improvement of our system.

Figure 10 presents a scenario where the vehicle turns right and the pedestrians cross the street in front of the car. The vehicle has a normal speed while moving, so the vehicle makes a fast approach to the pedestrians. As the car advances rapidly in the direction of the target, the standard tracking system switches the identification number associated with the detected pedestrian from *p4* to *p7* between the first and the second image. This problem appears as a result of the small size of the detection and the fast increase in scale. Our system correctly predicts that the detected person is the same one as in the first image. Moreover, in the third image, it can be noticed that our system maintains the same identification number for the person even after being occluded by the street light pole and after the change of the camera angle. We can observe that between the second and third image the standard tracking identifier changed from *p7* to *p20*.

Figure 11 is an example of a situation where the vehicle is moving to the left and the pedestrians are walking in two different directions on the sidewalk. In the initial image, we can observe the two pedestrians *p4* and *p2* going to the left and, respectively, to the right of the image. As the car is approaching the pedestrians and is turning left, pedestrian *p2* is wrongly re-identified in the second image by the standard tracking technique to *p11*, while our proposed system correctly assigns the same identifier. In the third image pedestrian *p4* is tracked correctly by our system and incorrectly by the standard system. The limitations in terms of scaling and angles of the standard tracking technique are overcome by our proposed method. What we can observe in this example is the way the trajectory predictor adapts depending on the movement of the vehicle. In the first image, the trajectory of the pedestrian *p4* is indicating that the pedestrian will move to the left of the image. The trajectory in the first image is generated based on observations made while the vehicle was moving straight. As the vehicle starts to turn left, in the second image the predictor combines the movement of the camera with the movement of the person and estimates that the position in the image of the person will remain the same. In the third image, we can observe that the predicted trajectory implies that the position of the pedestrian will be shifted to the right of the image, as the speed of the vehicle is higher than the speed of the pedestrian. The predicted trajectory correctly estimates the future positions of the person in the image, taking into consideration the movement of both vehicle and pedestrian.

Figure 12 presents the results of an experiment where the car is moving slower and is following a straight direction. The characteristic of this experiment is that the vehicle is moving slowly until the pedestrian crosses the street, then it starts to accelerate. As the pedestrians *p76* and *p77* are relatively close to the camera and there is no substantial movement of the vehicle, the standard tracking technique correctly re-identifies the pedestrians. When the pedestrians come in proximity to one another and the car starts moving a bit faster, which is the case in the third image, both people are assigned different identification numbers by the standard tracking method. Our proposed system is correctly re-identifying both pedestrians, maintaining their same identification numbers, with no identity switches.

To also have a legitimate testing phase based on standard tracking metrics, we computed the MOTA [45], IDF1 [46] and HOTA [47] values to prove the improvement of our system. We reported the metrics on the Caltech-Pedestrians dataset [42] using the same computation technique [44] used in Section 4.2.1. We generated both the ground truth data and the testing data based on the same bounding boxes extracted from the dataset, so the values of the metrics are strictly determined by the identification numbers assigned to the detections. The obtained values are reported in Table 6. The model uses the people trajectory predictor trained on the subsets s00 to s05 and is tested on the subsets s06 to s10. The dataset was pre-processed to simulate a lower framerate of 6 frames per second, for a more informed trajectory prediction.

The values reported in Table 6 demonstrate a significant improvement in our system when compared with the existing tracking technique. Our proposed system obtains greater values for all three metrics. This result is due to the complex nature of the people re-identification and tracking problem in the context of autonomous driving. In the case of autonomous driving, there are more complex factors that can impact the behaviour of the system than compared with the case of social robotics. To be able to have robust and reliable performance, the system needs to integrate changes in speed and direction of the camera, alongside appearance variations, such as scale and occlusions. While these elements are harder to predict by the standard tracking technique, our proposed system overcomes a considerable number of limitations and displays a satisfactory performance. The integration of a people trajectory prediction into the re-identification system produces a more informed and stable system.

#### 4.2.3. Validation in Bird’s-Eye View Scenarios

**Figure 13:**
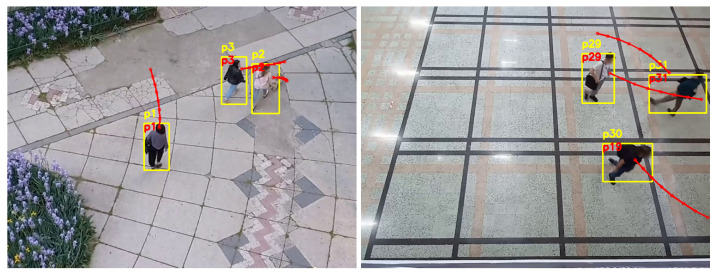
Person re-identification based on trajectory prediction in indoor and outdoor environments from a bird’s-eye point of view. The yellow ID represents the identification number estimated by the standard tracking technique, and the red ID represents the one generated by our system.

The previously presented scenarios assume that images are acquired from an eye-level point of view. Both a robotic platform and an autonomous vehicle would have the cameras placed to acquire images seen in perspective. The proposed system for person re-identification and tracking is also applicable for images acquired from a bird-eye point of view. Figure 13 shows an example of the performance of the re-identification system when applied to images captured from a bird’s-eye point of view, in both indoor and outdoor environments.

In Figure 13, the left image presents the result on an outdoor scene, while the right image presents the result on an indoor scene. The red dotted lines represent the predicted trajectories for each person in the image, after observing their previous positions. The advantage of processing images collected from a top view angle is that the people do not overlap while moving, so there are less occlusions and fewer cases of missing data. Moreover, the trajectory prediction system can have a more precise view of the interactions between the participants in the environment, manifesting better modelling of the behaviour of the people. In the right image of Figure 13 it can be noticed how the trajectories of person *p29* and *p31* are slightly curved, due to the social influence between the participants. This kind of performance of the trajectory prediction system would considerably improve the general functioning of the re-identification system when applied to bird’s-eye view images.

This validation scenario alongside the scenarios with social robotics and autonomous driving proves that the system can be utilised in a variety of applications. The system can be generalised and applied to any autonomous device. Moreover, the property of being suitable even for bird’s-eye view scenarios is important for applications where there are multiple streams of images coming from different angles. The information processed by the proposed system and combined from all the view angles should generate a stable and reliable people re-identification and tracking system.

## 5. Conclusions

The problem of person re-identification and tracking is a complex problem with large applicability in various domains. Autonomous devices rely on accurate results for safe and stable performance. In particular, social robots and self-driving cars depend on the performance of such a system for meaningful human-robot interactions and harmless navigation.

The person re-identification and tracking problem consists of the ability of a system to correctly recognise the same people over time in a sequence of images. For better results, tracking systems incorporate trajectory prediction methods to estimate the movement of the people. Trajectory prediction implies modelling the movement of an individual and predicting their next steps based on previous observations. The model behind the movement of a person needs to blend three pieces of information in order to obtain accurate results: the inner state of the person, social influence and scene understanding.

In this paper, we introduced a system for real-time people re-identification based on trajectory prediction. We designed a modular architecture for the re-identification part, composed of an object detector, a segmentation network, a person tracker and a trajectory predictor. For the trajectory predictor, we introduced a method that generates an image trajectory based on visual data and image coordinates. The semantic visual information associated with the scene is extracted using a semantic segmentation network. We use grid images to represent the movement of the individuals through the environment and the scene configuration. We pass the grids and the observed coordinates of the people to a generative adversarial network that incorporates a social pooling layer to generate the predicted trajectory.

The trajectory prediction system we introduced has two purposes: to improve the performance of a re-identification system and to anticipate the movement of the people, information that will help the autonomous platform to avoid collisions and safely navigate to a specific target. Our approach improves the capacity of re-identification as compared to other similar approaches by giving more reliable predictions. During the evaluation phase, the values obtained for the MOTA metric, for both social robotics and autonomous driving cases, demonstrate an improvement of over 5%.

We evaluated our approach individually for the introduced systems, people re-identification system and trajectory prediction component. We evaluated the performance of the systems on relevant existing datasets, with images acquired from the point of view of an autonomous platform, and on self-acquired data. We validated our approach in two different contexts: social robotics and autonomous driving. We integrated our work into the AMIRO robotic framework to validate our approach by improving the overall behaviour of a Pepper robot. In addition, we presented the applicability of the proposed system to scenarios where the images are acquired from a bird’s-eye point of view. The three settings that we used to verify our approach prove their suitableness for a variety of applications. We illustrated several scenarios from our experiments to demonstrate the behaviour of the proposed system. The presented method obtains more reliable re-identifications of the detected people, in more complex situations, overcoming problems such as occlusions, missing data or movement of the camera.

The proposed system deals with occlusions and missing detections by using the estimated motion of the people. The system can match one person that was not detected in a sequence of images by associating the estimated future position with the later detection. The system can integrate dynamic environments through the visual information about the scene, as the segmentation mask is computed for every input image. The continuously processed information also helps in the cases of moving cameras, as the system adjusts the trajectories based more on the recent data. The scaling problem is handled by the 2D coordinates representation of the people. Regardless of the size of the bounding box, the person is encoded by the center of the bounding box, making the system invariable to detection sizes. This approach, however, can present some problems with unstable people positions, considering the situations when the bounding boxes are not consistent and significantly vary in terms of detected area.

In the future, we are going to improve our work by including the influence of all the moving objects in the images, such as cars or animals. We further intend to extend the semantic information passed to the system, to include more relevant data, for more accurate re-identifications.

## Figures and Tables

**Table 1:** Values of ADE (Average Displacement Error) and FDE (Final Displacement Error) metrics for each subset in JRDB [39] dataset.

Scene Type	JRDB Subset	Pixel Coords	
		ADE	FDE
indoor	bytes-cafe_0	22.38	36.66
	clark-center_0	23.62	34.88
	cubberly-auditorium_0	42.96	65.64
	forbes-cafe_0	29.75	40.44
	gates-159-group-meeting_0	32.21	52.37
	gates-ai-lab_0	12.24	20.95
	gates-basement-elevators_1	42.54	69.33
	huang-2_0	26.46	39.48
	huang-basement_0	31.97	49.85
	jordan-hall_0	32.44	48.30
	nvidia-aud_0	30.53	45.81
	packard-poster-session_0	25.59	35.53
	packard-poster-session_1	7.77	13.61
	packard-poster-session_2	4.61	6.45
	stlc-111_0	21.72	38.18
	svl-meeting-gates-2_0	15.22	23.62
	svl-meeting-gates-2_1	16.08	25.39
	tressider_2	15.07	21.67
outdoor	clark-center_1	35.77	53.31
	clark-center-intersection_0	24.19	34.64
	gates-to-clark_1	27.45	33.03
	hewlett-packard-intersection_0	10.60	20.24
	huang-lane_0	21.97	40.58
	memorial-court_0	29.97	36.70
	meyer-green_0	29.78	49.26
	tressider_0	17.23	28.76
	tressider_1	9.71	16.70
	Average error	23.69	36.34

**Table 2:** Comparison considering ADE (Average Displacement Error) and FDE (Final Displacement Error) metrics on UCY [40] and ETH [41] datasets on existing systems.

Metric	Dataset	S-LSTM [24]	S-GAN [25]	SoPhie [28]	Ours
ADE	ZARA 1 [40]	0.27/0.47	0.21/0.34	__ /0.30	0.29/0.45
	ZARA 2 [40]	0.33/0.56	0.27/0.42	__ /0.38	0.33/0.55
	Students03 [40]	0.41/0.67	0.36/0.60	__ /0.54	0.96/1.05
	Hotel [41]	0.49/0.79	0.48/0.72	__ /0.76	1.57/1.47
	ETH [41]	0.73/1.09	0.61/0.81	__ /0.70	2.38/1.92
FDE	ZARA 1 [40]	0.56/1.00	0.42/0.69	__ /0.63	0.55/0.91
	ZARA 2 [40]	0.70/1.17	0.54/0.84	__ /0.78	0.63/1.12
	Students03 [40]	0.84/1.40	0.75/1.26	__ /1.24	1.60/1.91
	Hotel [41]	1.01/1.76	0.95/1.61	__ /1.67	2.73/2.62
	ETH [41]	1.48/2.35	1.22/1.52	__ /1.43	4.11/3.03

**Table 3:** Values of ADE (Average Displacement Error) and FDE (Final Displacement Error) metrics for each subset in Caltech-Pedestrians [42] dataset.

Caltech-Pedestrians Subset	Pixel Coords	
	ADE	FDE
set00	11.05	21.35
set01	7.19	13.17
set02	6.70	11.73
set03	9.68	17.79
set04	5.61	9.74
set05	7.40	13.85
set06	4.20	7.17
set07	7.03	12.82
set08	4.42	7.20
set09	7.01	13.52
set10	6.80	13.10
Average error	7.00	12.85
Default split [42]	6.29	11.78

**Table 4:** Tracking metrics values for the trajectory-based re-identification system on MOT17 dataset [43].

Metric	Standard Tracking	Proposed System
MOTA [45] (%)	55.05	61.04
IDF1 [46] (%)	55.62	52.24
HOTA [47] (%)	45.57	43.79

**Table 5:** Mean processing time of the trajectory-based re-identification system considering different numbers of tracked people.

Number of Tracked People	Proposed System (Milliseconds)
1	102.06
2	106.55
3	106.63
4+	108.22

**Table 6:** Tracking metrics values for the trajectory-based re-identification system on Caltech-Pedestrians dataset [42].

Metric	Standard Tracking	Proposed System
MOTA [45] (%)	89.22	94.92
IDF1 [46] (%)	78.36	90.10
HOTA [47] (%)	78.96	89.15

## Data Availability

Not applicable.
